# The effect of antiplatelet therapy and oral anticoagulants on the accuracy of faecal immunochemical testing

**DOI:** 10.1308/rcsann.2024.0015

**Published:** 2024-03-13

**Authors:** F Wu, AA Khan, M Klimovskij, R Harshen

**Affiliations:** ^1^ East Sussex Hospitals NHS Trust, UK; ^2^ East Kent Hospitals University NHS Trust, UK

**Keywords:** Colorectal cancer, Screening, Anticoagulation

## Abstract

**Introduction:**

Faecal immunochemical testing (FIT) has been adopted to identify patients requiring further investigations on the colorectal cancer (CRC) referral pathway. We aimed to investigate the effect of antiplatelet and anticoagulant drugs on the accuracy of FIT results.

**Methods:**

This observational study categorised patients with suspected CRC symptoms, who completed both FIT and colonic investigations, into two groups (control and exposed) based on their use of antiplatelet and anticoagulant drugs. Two-by-two tables and receiver operating characteristic (ROC) curve analysis were used to determine accuracy.

**Results:**

A total of 928 patients were divided into a control (*n*=683) and an exposed group (*n*=245). A nonsignificant higher proportion of patients tested positive in the exposed group (24.1% vs 18.4%, *p*=0.063). For detection of CRC, improved sensitivity of 87% vs 81.2%, specificity of 84.8% vs 79.9% and negative predictive value of 99.2% vs 98.3% was calculated in the control vs exposed groups, respectively. The positive predictive value was comparable between the two groups (21.4% vs 22% in the control and exposed groups, respectively). In ROC analysis, there was no difference between the groups (AUC 90% vs 87%, *p*=0.56). The use of antiplatelet and anticoagulant drugs did not increase the risk of positive FIT results on multivariate logistic regression analysis.

**Conclusions:**

FIT accuracy for CRC detection remained unaffected despite more patients testing positive in the exposed group. FIT should be considered a supplementary tool for triage. Antiplatelet and anticoagulant drugs do not need to be discontinued before collection of FIT.

## Introduction

The detection of blood in faeces remains the most effective way to screen for colorectal cancers (CRC).^1^ Faecal immunochemical testing (FIT) analysers deploy immunoglobulin directed against human haemoglobin, enabling detection and quantification of minute amounts of faecal blood.^2^ FIT is very specific for predicting lesions in the colon.^3^ The diagnostic accuracy of FIT as an aid for detecting CRC has demonstrated a high negative predictive value (NPV >99%), making FIT a reliable rule-out test.^4^ There is a transition from guaiac-based faecal occult blood tests (FOBt) to FIT in the UK bowel cancer screening programme.^5^ The use of FIT may also identify high-risk symptomatic patients referred on a two-week wait pathway and help streamline investigations appropriately.^6^

Lower gastrointestinal bleeding resulting from nonmalignant disease processes has the potential to elevate false-positive rates for FIT.^7^ This may lead to unnecessary investigations and increased healthcare expenditure.^8^ The use of antiplatelet and anticoagulant drugs can increase the risk of gastrointestinal bleeds.^9,10^ Several studies have identified that the use of nonsteroidal anti-inflammatory drugs, such as antiplatelet therapy and oral anticoagulants, is associated with false-positive FIT results.^11,12^ Current European and American guidelines do not recommend the discontinuation of these medications before the collection of faecal samples for FIT.^13,14^ However, limited data are available on the impact of new generation direct oral anticoagulants (DOACs) on the performance of FIT results.

Prescriptions for antiplatelet therapy and oral anticoagulation are rising among individuals aged 65 years and older.^15^ These medications play a crucial role in preventing the risk of myocardial infarction, stroke and venous thromboembolism. The higher prevalence of these medical conditions in the aging population, coupled with the growing diagnosis of cancer among the elderly, highlights the need to understand the effect of antiplatelet and anticoagulant drugs on FIT.^16,17^ CRC exhibits the highest incidence among octogenarians in the UK, underscoring the significance of these medications in addressing the complex health needs of the elderly.^18,19^

This study was designed to investigate the effects of antiplatelet therapy and oral anticoagulation, including the new generation DOACs, on FIT results in a large cohort of symptomatic patients. Results of this study involve gathering evidence from real-world examples. This will in turn support clinicians with interpretation of FIT results in this specific cohort of patients. The primary aim of the study was to determine whether the use of antiplatelet therapy and oral anticoagulation would affect the accuracy of FIT for CRC. The secondary aim was to establish the role of these individual medications on the false-positive rate of FIT results.

## Methods

### Study sample and design

This observation study was conducted from 1 August 2017 to 31 December 2018 at two district general hospitals in England. A target sample size was set at 1,000 patients. This was based on the research objectives, effect size and practical considerations of the study. The study was not statistically powered.

**Figure 1:**
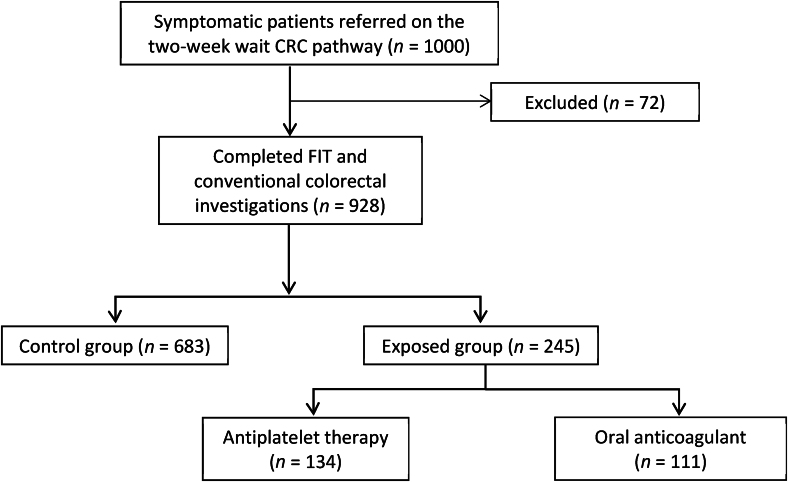
Flow of participants through the study. CRC = colorectal cancer; FIT = faecal immunochemical testing.

The inclusion criteria for the study included all consecutive adult patients (≥18 years of age) who were referred on a two-week-wait pathway with symptoms suspected of CRC, had returned a FIT result and had undergone colorectal investigations. These referrals were made either by their primary care provider or through the national bowel cancer screening programme. The National Institute for Health and Care Excellence (NICE) and the Association of Coloproctology of Great Britain and Ireland (ACPGBI) have recommended that patients with suspected CRC should be offered either a colonoscopy or computed tomography (CT) colonography as the choice of investigations.^5,20^ The flow of participants through the study is shown in Figure 1.

The exclusion criteria consisted of incomplete FIT results, the presence of obvious rectal bleeding at the time of stool collection and patient refusal to proceed with colonoscopy or CT colonography. In all, 72 patients were excluded from the study. A total of 928 patients returned full results and were included in the final analysis. These patients were divided into either the ‘control’ or ‘exposed’ group, based on their use of any antiplatelet or anticoagulant drug.

### FIT sampling and analysis

All participants were required to give consent for rectal examination and FIT. A small quantity of faecal sample was collected by a medical practitioner during digital rectal examination in the surgical outpatient clinic. The faecal sample was immediately smeared on an EXTEL HEMO-AUTO MC Collection Picker device (Kyowa Medex, Japan) and preserved according to the manufacturer's guidelines. All samples were transported to the hospital laboratory for analyses the following day.

The HM-JACKarc automated analyser system (Kyowa Medex, Japan and Alpha Laboratories, UK) was used to quantify faecal haemoglobin (f-Hb). The f-Hb measurements were reported as micrograms Hb per gram of faeces. The minimum and maximum reported values were 0.0 and >450μg Hb/g faeces, respectively. NICE and ACPGBI have recommended a threshold value of ≥10μg Hb/g faeces as the criterion for determining a positive result.^5,21^ The analysis was carried out by a registered biomedical scientist and the results authorised by two consultant clinical scientists before the issue of results. The laboratory has a comprehensive quality management system and is accredited to ISO 15189-based standards.

### Data collection and statistical analysis

Prospective data, which included symptoms, FIT results, outcomes of investigations and final diagnoses, were collected. The distribution of f-Hb was checked for normality with Shapiro-Wilk test and Q-Q plots and showed nonparametric distribution. The Mann-Whitney *U* test was used to compare qualitative variables and the chi-squared test was used to compare categorical variables between the two groups. Receiver operator curve (ROC) analysis and area under the curve (AUC) estimates were used to determine and compare the diagnostic accuracy of FIT in detecting CRC between the two (control vs exposed) groups. Two-by-two tables were used to calculate sensitivity, specificity, positive and negative predictive values (PPV, NPV) and odds ratios (OR). Binary logistic regression analysis was used to calculate the predictive values of each variable on the FIT results.

Priori subgroup analysis by individual drugs was performed: for the purpose of these analyses, patients taking dipyridamole (*n*=2) and a combination of aspirin and dipyridamole (*n*=1) were included in the ‘aspirin’ group and patients taking a combination of aspirin and clopidogrel (*n*=7) were included in the ‘clopidogrel’ group. The details of each antiplatelet therapy and oral anticoagulant taken by the participants are shown in Supplementary Table 1. Any differences were reported with 95% confidence intervals (CI). *p*<0.05 was statistically significant. Data maintenance and analyses were performed using Microsoft Excel® (2013) and IBM® SPSS® version 26 (SPSS Inc, Chicago, IL, US).

## Results

A total of 928 individuals returned a FIT result and completed either a colonoscopy or CT colonography. The demographic characteristics of the patients are summarised in Table 1. The 928 patients were divided into either the ‘control’ (*n*=683, Male: Female=1:2, median age 70 years; interquartile range (IQR) 62–78) or ‘exposed’ (*n*=245, Male: Female=1:1, median age 77 years, IQR 72–82) group. Patients in the control group were younger than in the exposed group (*p*<0.001), with a greater number of females than males (*p*=0.008).

**Table 1:** Demographic characteristics of study participants

Demographic characteristics	Control	Exposed*	*p*-value^‡^
Total patients	683 (73.6%)	245 (26.4%)	
Male	259 (37.9%)	117 (47.8%)	0.008
Female	424 (62.1%)	128 (52.2)	
Median age (IQR)	70 (62–78)	77 (72–82)	<0.001^§^
FIT results			
FIT negative	557 (81.6%)	186 (75.9%)	0.063
FIT positive	126 (18.4%)	59 (24.1%)	
Results of colonic investigations in symptomatic patients			
Colorectal cancer	31 (4.5%)	16 (6.5%)	0.236
High-risk polyps^†^	22 (3.2%)	8 (3.3%)	1.0
Colitis	36 (5.3%)	5 (2%)	0.044
Diverticulosis	164 (24%)	73 (29.8%)	0.087
Haemorrhoids	25 (3.7%)	4 (1.6%)	0.137
Other diagnosis	174 (25.4%)	71 (28.9%)	0.285
Normal	231 (33.8%)	68 (27.8%)	0.094

FIT = faecal immunochemical testing; IQR = interquartile range.

*Patients taking either antiplatelets or oral anticoagulants.

†Polyp size ≥10mm or polyp count >5.

‡Chi-square test.

^§^Mann-Whitney *U* test.

In the exposed group, 134 (54.6%) patients were taking antiplatelet therapies and 111 (45.3%) patients were taking oral anticoagulants (Figure 1). A higher proportion of patients in the exposed group had a positive result for FIT (24.1% vs 18.4%), but this was not statistically significant (*p*=0.063). The prevalence of CRC was similar in both groups (6.5% vs 4.5%, *p* 0.236). The proportion of colitis was statistically significantly lower in the exposed group (2% vs 5.3%, *p*=0.044), whereas the proportion of diverticulosis was higher (29.8% vs 24%, *p*=0.087). The exposed group also had a lower proportion of normal colorectal investigations (27.8% vs 33.8%, *p*=0.094; Table 1).

The accuracy of FIT for CRC was slightly worse in the exposed group, with a sensitivity and specificity of 81.2% and 79.9%, compared with 87% and 84.8% in the control group, respectively. The false-positive rates and PPV were similar in both groups (77.9% vs 78.5%, and 22% vs 21.4%, in the exposed vs controls, respectively). On subgroup analysis, the PPV was 26.6% and 17.2% for patients taking antiplatelet and anticoagulant drugs, respectively.

There was an increase in the false-negative proportions (1.6% in the exposed vs 0.7% in the controls), lowering the NPV to 98.3% in the exposed group (Table 2). In subgroup analyses, patients taking oral anticoagulation, particularly rivaroxaban, frequently tested positive on FIT analysis (OR 2.3, 95% CI 1.1–4.7, *p*=0.02; Table 3). In multivariate logistic regression analysis, neither antiplatelet therapy nor oral anticoagulation usage increased the risk of positive FIT results (Table 4).

**Table 2:** Diagnostic accuracy of FIT for CRC in control and exposed groups

	Sensitivity	Specificity	PPV	False-positives	NPV	False-negatives
Control	87% (69.2–95.7%)	84.8% (81.7–87.4%)	21.4% (14.8–29.8%)	78.5% (70.1–85.1%)	99.2% (98–99.7%)	0.7% (0.2–1.9%)
Exposed	81.2% (53.6–95%)	79.9% (74–84.7%)	22% (12.6–35%)	77.9% (64.9–87.3%)	98.3% (94.9–99.5%)	1.6% (0.4–5%)
Antiplatelet therapy	88.8% (50.6–99.4%)	82% (74.3–88.4%)	26.6% (12.9–46.1%)	73.3% (53.8–87%)	99% (93.9–99.9%)	0.9% (0.05–6%)
Oral anticoagulant	71.4% (30.2–94.8%)	76.9% (67.4–84.3%)	17.2% (6.5–36.4%)	82.7% (63.5–93.4%)	97.5% (90.6–99.5%)	2.4% (0.42–9.3%)

CRC = colorectal cancer; FIT = faecal immunochemical testing; NPV = negative predictive value; PPV = positive predictive value.

**Table 3:** The positive and negative rates of FIT (for CRC) for each drug

	Faecal immunochemical test			
	Positive (%)	Negative (%)	OR (95% CI)	*p*-value*
Antiplatelet	30 (22.4%)	104 (77.6%)	1.2 (0.81–1.9)	0.33
Aspirin	24 (22.6%)	82 (77.4%)	1.2 (0.78–2.1)	0.35
Clopidogrel	9 (23.1%)	30 (76.9%)	1.3 (0.61–2.8)	0.52
Anticoagulant	29 (26.1%)	82 (73.9%)	1.5 (0.98–2.4)	0.07
Warfarin	12 (23.1%)	40 (76.9%)	1.3 (0.67–2.6)	0.46
Apixaban	5 (26.3%)	14 (73.7%)	1.5 (0.55–4.4)	0.55
Rivaroxaban	12 (34.3%)	23 (65.7%)	2.3 (1.1–4.7)	0.02

CI = confidence interval; CRC = colorectal cancer; FIT = faecal immunochemical testing; OR = odds ratio.

*Fisher exact probability test (two-tailed).

**Table 4:** The comparative ROC analyses for FIT for colorectal cancer between the control and exposed groups

Factor	OR (95% CI)	*p*-value
Age	1.005 (0.989, 1.021)	0.549
Gender	0.683 (0.477, 0.979)	0.038
CRC	0.031 (0.014, 0.072)	<0.001
Colitis	0.283 (0.144, 0.559)	<0.001
High-risk polyps*	0.386 (0.174, 0.857)	0.019
Aspirin	0.79 (0.455, 1.369)	0.400
Clopidogrel	1.033 (0.425, 2.513)	0.942
Warfarin	0.746 (0.355, 1.565)	0.438
Apixaban	0.619 (0.199, 1.918)	0.405
Rivaroxaban	0.484 (0.217, 1.08)	0.076

CI = confidence interval; FIT = faecal immunochemical testing; OR = odds ratio; ROC = receiver-operator curve.

*Polyp size ≥10mm or polyp count >5.

**Figure 2:**
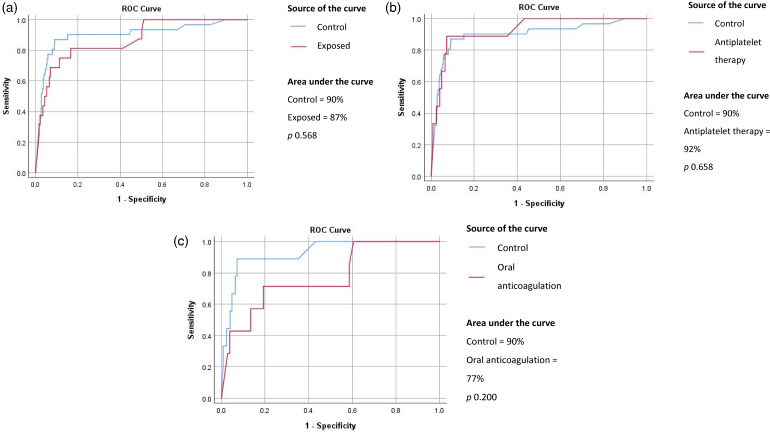
ROC analysis between (a) control and exposed groups, (b) control group and patients taking antiplatelet therapy and (c) control group and patients taking oral anticoagulants. ROC = receiver operator curve.

In ROC analysis, there was a nonsignificant decrease in the AUC from 90% in the control to 87% in the exposed group (*p*=0.568). In subgroup analyses, patients on antiplatelet therapy had a better AUC (92%) and patients on oral anticoagulation had the worst AUC (77%) compared with controls (90%; Figure 2a–c).

## Discussion

FIT has gained widespread acceptance as a screening tool for CRC.^20^ While antiplatelet and anticoagulant drugs are prescribed frequently for the prevention of thrombosis in cardiovascular and cerebrovascular diseases, their use is associated with an increased risk of gastrointestinal bleeding.^21^ In our investigation, FIT accuracy for CRC detection remained generally reliable when concurrently taken with antiplatelet therapy or oral anticoagulation. Findings from this study are supported by the results of previous work.^22^

A comparative study by Tsuji *et al* reported a similar detection rate of CRC between controls and users of DOACs (5.8% vs 5.8%, *p*=1.0), advanced neoplasm (19.3 vs 19.4%, *p*=1.0) or any neoplasm (48.4% vs 47.5%, *p*=0.85).^23^ A meta-analysis by Nieuwenburg *et al* showed no statistical difference in the PPV for CRC between users of either aspirin or oral anticoagulants and nonusers.^24^ A post hoc analysis of participants assigned to the FIT arm of a randomised controlled study reported a higher frequency of FIT-positive results in patients taking oral anticoagulants, without showing a significant difference in PPV for advanced neoplasia of the colon.^25,26^ The authors of that study, Bujanda *et al*, did not perform colonoscopies on individuals with FIT-negative results; therefore, they were unable to determine the NPV of FIT.

In our study population, an uneven distribution of patients was observed between both groups. This discrepancy reflects the demographics of our patient cohort, where most are not prescribed antiplatelet therapy or oral anticoagulants. Additionally, patients in the exposed group were older with potential frailty and comorbidities. These factors may have influenced their decision not to proceed with invasive investigations, due to concerns about the associated risks.^27^ Furthermore, we observed an increased prevalence of abnormal colonic findings in the exposed group, aligning with national statistics on CRC.^18,19^

In this study, ROC analysis revealed no significant difference in the AUC between the control and exposed groups when users of antiplatelet and anticoagulant drugs were pooled in the exposed group (Figure 2a). In subgroup analyses, there was a marginal improvement in AUC for patients taking antiplatelet therapy compared with the control group (92% vs 90%, *p*=0.658; Figure 2b). Previous studies have suggested that aspirin usage could enhance FIT sensitivity for CRC; however, a recent randomised control trial failed to prove any effects of aspirin on the performance of FIT.^28–30^ Conversely, a decline in AUC was noted for users of oral anticoagulants compared with nonusers in our study (77% vs 90%, *p*=0.200; Figure 2c). The reduction in AUC observed in users of oral anticoagulants may be attributed to the inclusion of patients taking new-generation DOACs, such as rivaroxaban. A meta-analysis showed increased odds of gastrointestinal bleeding with rivaroxaban compared with conventional oral anticoagulants.^7^

Rivaroxaban has been associated with a higher rate of FIT-positive results, potentially by facilitating bleeding from nonmalignant colorectal lesions, thereby increasing false-positive rates. In our analysis, the subgroup of patients taking rivaroxaban was at increased risk of testing positive on FIT analysis (OR 2.3, 95% CI 1.1–4.7, *p*=0.02). Other studies have reported increased positivity rates with the use of other anticoagulants, such as acenocoumarol (9.3% vs 6.2% in controls) and dual antiplatelet therapy (22.2% vs 6.3% in controls).^7,25,26^ Notably, the overall FIT positivity was not affected by rivaroxaban when other factors were considered in multivariate logistic regression analysis (OR 0.48, 95% CI 0.21–1.08, *p*=0.076; Table 4). Our study identified several variables, such as male gender, CRC, colitis, and high-risk polyps as the best predictors of positive FIT results (Table 4).

It is important to acknowledge that the accuracy of FIT is dependent on the sample size and disease prevalence in each group. Although there was a nonsignificant increase in the proportion of patients testing positive on FIT in users of antiplatelet therapy and oral anticoagulants, no significant differences were observed in the false-positive rates (Table 2). This finding could be attributed to the increased prevalence of CRC in the exposed group (see below). However, when conducting subgroup analyses, the PPV was notably lower among users of oral anticoagulants (17.2% vs 21.4% in the control group). In contrast, users of antiplatelet therapy exhibited a higher PPV (26.6% vs 21.4% in the control group). This discrepancy is due to the lower prevalence of CRC in users of oral anticoagulants and a higher prevalence of CRC in the antiplatelet therapy group.

Randel *et al* conducted a cross-sectional study in a substantial patient cohort as part of a Norwegian CRC screening trial.^12^ The authors reported a significantly lower PPV for CRC detection in users of DOACs (0.9% vs 6.8% in controls, *p*=0.001) after matching each group for age, sex, screening centre and screening round. The remarkably low PPV observed in DOAC users could be attributed to the lower-than-expected prevalence of CRC in that demographic. In their investigation, CRC was identified in only two of the 212 individuals using DOACs, while our study identified CRC in 16 of the 245 patients in the exposed group. Additionally, our study population differed from that of the Norwegian study. The Norwegian researchers focused exclusively on individuals in the screening age range of 50–74 years, while our study encompassed all adult patients referred to the suspected CRC clinic. Consequently, our study population was older than the participants in the Norwegian study.

As highlighted earlier, the incidence of CRC is associated with advancing age. This age-related distinction may account for the higher prevalence of CRC observed in our cohort of patients.

In this study, a reduction in NPV in the exposed group was observed (98.3% vs 99.2% in controls), particularly among users of oral anticoagulants (97.5% vs 99.2% in controls). This is an important finding in the context of bowel cancer screening. FIT is used as a triage rule-out test in the detection of CRC. A reduction in FIT accuracy, specifically a false-negative FIT result, can lead to false reassurances and may delay the diagnosis of CRC.

In the Nottingham service evaluation, the authors observed that individuals with negative FIT results were more likely to have cancer at alternative sites.^31^ Chapman *et al* identified a modest, yet significant, proportion of noncolonic malignancies in patients with negative FIT results. The detection rate for cancers at alternative sites was higher among FIT-negative patients compared with those with FIT-positive results (5.1% vs 2.4%, chi-square 3.9, *p*<0.05). Thus, healthcare professionals are advised to interpret negative FIT results with caution in users of oral anticoagulants and to consider additional investigations beyond the colon.

## Strengths and limitations

This study has several strengths. Firstly, the data collection process was conducted prospectively, eliminating potential recall bias. Secondly, a clinician collected the faecal samples for FIT during digital rectal examination, ensuring full compliance. Thirdly, there are limited published data on the effects of new generation DOACs on the performance of FIT for CRC in symptomatic patients, making our study a valuable addition to the literature. Fourthly, all patients underwent colorectal investigations regardless of the FIT result, allowing for the determination of the effects of these drugs on both positive and negative predictive values. Lastly, our inclusion of all patients referred to the suspected CRC clinic provides a broader and more comprehensive representation of the diverse age groups affected by CRC.

This study has several limitations. Firstly, the study population was not randomised, potentially introducing biases into the results. Secondly, the two groups were not matched, which could affect the comparability of the results. Lastly, the condensed sample size in the exposed group during subgroup analysis may affect the precision of the conclusions drawn from the analysis. Recent investigations into the impact of other variables on the false-positivity rate of FIT results have revealed that demographics, such as social deprivation, and the use of other medications, such as proton pump inhibitors, can contribute to false-positive results.^32–35^ To address these limitations, a complex randomised control study design with a large elderly patient population would be required. Additionally, matching criteria for age, gender, deprivation, medications, and comorbidities would need to be applied to enhance the robustness of the study design and mitigate potential confounding factors.

## Conclusions

In conclusion, FIT performance is generally reliable in patients taking antiplatelet and anticoagulant medications. However, results from previous studies highlight the complexity of this relationship. Our findings emphasise the importance of understanding the nuances of FIT performance in specific patient populations. FIT should be considered a supplementary tool in current assessment approaches, and a negative FIT result in individuals using anticoagulant drugs may warrant careful interpretation, necessitating a low threshold for clinical review and further investigations. Based on the present literature, we recommend that antiplatelet and anticoagulant drugs do not need to be discontinued before collection of faecal sampling for FIT.

## Ethics statement

Ethics approval was not required for this study. There was strict adherence to research governance guidelines throughout this study. This study was carried out under the Code of Ethics of the World Medical Association (Declaration of Helsinki).
